# Comment on: Comparison of carbapenem MIC for NDM-producing Enterobacterales by different AST methods: considerations for media composition

**DOI:** 10.1093/jacamr/dlae091

**Published:** 2024-06-11

**Authors:** Tomefa E Asempa, David P Nicolau

**Affiliations:** Center for Anti-Infective Research and Development, Hartford Hospital, Hartford, CT, USA; Center for Anti-Infective Research and Development, Hartford Hospital, Hartford, CT, USA; Division of Infectious Diseases, Hartford Hospital, Hartford, CT, USA

We read with great interest the recent article by Lee *et al*.^1^ on the performance of the MIC test strip (ETEST), automated AST card (Vitek 2) and Sensititre broth microdilution (BMD) in determining carbapenem susceptibility and MIC values for NDM-producing Enterobacterales. We applaud the authors for providing further evidence of suboptimal test performance of these challenging-to-treat bacteria across several clinically utilized susceptibility testing modalities. However it is unfortunate that this study did not evaluate or acknowledge the impact of an important contributor to this variability, namely zinc concentrations in each medium.

A number of studies over several decades have shown that the type of medium can have a profound effect on MICs and susceptibility patterns of bacteria based on (i) species (e.g. *Pseudomonas aeruginosa*), (ii) resistance mechanism (e.g. MBL) and (iii) mechanism of action of the antimicrobial agent (e.g. colistin/polymyxin B, daptomycin, tigecycline, cefiderocol).^2–7^

Unlike the requirement for cation-adjusted Mueller–Hinton broth to have specific concentration ranges for calcium and magnesium, the concentrations of zinc are not specified or regulated. Therefore, for a study to assess performance or compare β-lactam susceptibility of MBL-producing isolates between tests that involve different media, consideration of zinc concentrations should be included to contextualize the study results. In this case, the dependence of MBL hydrolysis on zinc cations can lead to variability in MICs based on differences in the zinc concentrations in the testing media. Indeed, up to 10-fold differences in zinc concentrations have been observed in the broth of several automated susceptibility testing platforms (Vitek, MicroScan, BD Phoenix and Sensititre), whereas 4-fold differences in zinc concentrations have been observed in commercial broth routinely used for manual broth microdilution (Becton Dickinson, Oxoid and Sigma-Aldrich).^8,9^

In addition, we have shown that different lots of Mueller–Hinton broth even from the same manufacturer can result in different MICs and susceptibility categories of MBL-harbouring Enterobacterales.^9^ This latter finding could also be relevant to the study by Lee *et al.*, given that the details of the Muller–Hinton agar (MHA) plates on which the Etests were placed for testing were not provided. If several brands or the same brand but different lots were indeed used that can also introduce variability in assessing Etest assay performance as it has been shown that the concentrations of cations in MHA also vary.^3,10^

In conclusion, differences in testing performance across media types and lots is not unexpected when MBLs are involved, and to further elucidate sources of variability, future studies would need to examine media and cation composition.

## References

[1] Lee ALH, Leung ECM, Chow VCY. Comparison of carbapenem MIC for NDM-producing Enterobacterales by different AST methods. JAC Antimicrob Resist 2024; 6: dlae028. 10.1093/jacamr/dlae028 38686026 PMC11057202

[2] Barry AL, Reller LB, Miller GH et al Revision of standards for adjusting the cation content of Mueller-Hinton broth for testing susceptibility of *Pseudomonas aeruginosa* to aminoglycosides. J Clin Microbiol 1992; 30: 585–9. 10.1128/jcm.30.3.585-589.1992 1551973 PMC265114

[3] Girardello R, Bispo PJM, Yamanaka TM et al Cation concentration variability of four distinct Mueller-Hinton agar brands influences polymyxin B susceptibility results. J Clin Microbiol 2012; 50: 2414–8. 10.1128/JCM.06686-11 22553247 PMC3405622

[4] Fernández-Mazarrasa C, Mazarrasa O, Calvo J et al High concentrations of manganese in Mueller-Hinton agar increase MICs of tigecycline determined by Etest. J Clin Microbiol 2009; 47: 827–9. 10.1128/JCM.02464-08 19144806 PMC2650928

[5] Huband MD, Ito A, Tsuji M et al Cefiderocol MIC quality control ranges in iron-depleted cation-adjusted Mueller-Hinton broth using a CLSI M23-A4 multi-laboratory study design. Diagn Microbiol Infect Dis 2017; 88: 198–200. 10.1016/j.diagmicrobio.2017.03.011 28410852

[6] Fuchs PC, Barry AL, Brown SD. Daptomycin susceptibility tests: interpretive criteria, quality control, and effect of calcium on in vitro tests. Diagn Microbiol Infect Dis 2000; 38: 51–8. 10.1016/S0732-8893(00)00164-4 11025184

[7] Simon M, Gerlach RG, Pfeifer Y et al Increased zinc levels facilitate phenotypic detection of ceftazidime-avibactam resistance in metallo-β-lactamase-producing gram-negative bacteria. Front Microbiol 2022; 13: 977330. 10.3389/fmicb.2022.977330 36483203 PMC9723239

[8] Asempa TE, Gill CM, Chibabhai V et al Comparison of zinc concentrations in the broth of commercial automated susceptibility testing devices (Vitek 2, MicroScan, BD Phoenix, and Sensititre). Microbiol Spectr 2022; 10: e0005222. 10.1128/spectrum.00052-22 35377221 PMC9045177

[9] Bilinskaya A, Buckheit DJ, Gnoinski M et al Variability in zinc concentration among Mueller-Hinton broth brands: impact on antimicrobial susceptibility testing of metallo-β-lactamase-producing Enterobacteriaceae. J Clin Microbiol 2020; 58: e02019-20. 10.1128/JCM.02019-20 32999009 PMC7685897

[10] Humphries RM, Ambler J, Mitchell SL. et al CLSI Methods Development and Standardization Working Group best practices for evaluation of antimicrobial susceptibility tests. J Clin Microbiol 2018; 56: 10.1128/JCM.01934-17 PMC5869819 29367292

